# Differential responses of soil C, N, and P ecological stoichiometric characteristics to different configurations of edge-locked forests in the Kubuqi Desert

**DOI:** 10.3389/fpls.2025.1520024

**Published:** 2025-01-30

**Authors:** Xue Chen, Hejun Zuo, Min Yan, Haibing Wang, Cheng Xi, Yinchao Chai

**Affiliations:** ^1^ Inner Mongolia Key Laboratory of Aeolian Physics and Desertification Control Engineering, College of Desert Control Science and Engineering, Inner Mongolia Agricultural University, Hohhot, China; ^2^ Hanggin Desert Ecosystem Positioning Research Station, Ordos, China

**Keywords:** desertification, edge-locked forests, C, N, and P, ecological stoichiometry, particle size characterization, Kubuqi Desert

## Abstract

As a vital component of the desert ecological protection system, the edge-locked forests of the Kubuqi Desert play a crucial role in mitigating wind erosion, stabilizing sand, maintaining soil and water, and restricting desert expansion. In this paper, six types of standard protection forests in the Kubuqi Desert, namely *Salix psammophila* (SL), *Elaeagnus angustifolia* (SZ), *Salix matsudana* (HL), *Corethrodendron fruticosum+Salix psammophila* (YC + SL), *Caragana korshinskii + Populus simonii* (XYY + NT), and *Elaeagnus angustifolia + Salix matsudana* (SZ + HL), were investigated. Notably, the vertical differentiation patterns of soil carbon (C), nitrogen (N), phosphorus (P), and ecological stoichiometric ratios, as well as soil particle size features within the 0–100-cm soil layer under protection forests with different allocation modes, were systematically and comprehensively analyzed. The study’s findings showed that: (1) Among the six configuration types, SZ, NT + XYY, and SL exhibited higher soil SOC and TN concentrations. Both soil SOC and TN content decreased with increasing soil depth, whereas soil TP content displayed no considerable variation among different stand types or soil depths. (2) Based on the N/P threshold hypothesis, N was the limiting nutrient element for the growth of edge-locked forests in the region. (3) The understory soils of different configurations of edge-locked forests mainly comprised sand. The silt and clay contents of SL and NT + XYY were substantially higher than those of the other four configurations. The vertical distribution patterns of particle size and parameter characteristics had variations. (4) Soil C, N, P, and stoichiometric characteristics are affected by vegetation type, soil depth, and soil texture. In conclusion, SZ and SL can be used as the dominant tree species in the edge-locked forests of the Kubuqi Desert, and the NT + XYY mixed forest configuration pattern displays the most apparent soil improvement effect. This study’s findings offer a scientific reference and foundation for restoring vegetation and enhancing the ecological environment in desert regions. In addition, they provide a theoretical foundation for establishing and managing edge-locked forests.

## 1 Introduction

Desertification is one of the biggest global environmental issues (Rivera-Marin et al., 2022). It exploits one-third of the worldwide land mass and is a prominent geochemical cycle component (Chen H. et al., 2024). However, climate change is primarily responsible for increasing the scope of desertification around the world. The latest data from the United Nations (the 2022 national reports of 126 Member States) show that 15.5 percent of the earth’s land is already degraded, an increase of 4 percent over the past few years. The area ravaged by desertification has grown from 50,000 to 70,000 square kilometers annually, especially in regions near deserts (Kumari et al., 2023). Desert expansion destroys the biological potential of the land (Stiles, 1995) while causing a loss of ecological stability, a decline in biodiversity, and a reduction in carbon (C) sink capacity (Gupta, 2012). It threatens human survival, adult production, and life (Zhang et al., 2017). Therefore, effective desert expansion prevention is strategically significant for ensuring ecological security and realizing sustainable development in arid areas (Chen L. et al., 2024). According to pertinent research, vegetative measures seem to be the most effective approach to break off the spread of deserts (Wu et al., 2023). Essentially, edge-locking forests are one of the most standard and effective methods of preventing desert expansion. Desert edge-locked forests present a broad range of services by effectively reducing wind speeds, blocking sand particles, and curbing the spread of deserts. Meanwhile, edge-locked forests can maintain soil and water, refine soil, increase ground vegetation cover, regulate climate, and conserve water, which not only can effectively alleviate the deterioration of the regional ecological environment but is also crucial for the development of sustainable local desert ecosystems in terms of economy, society, and culture (Jenkins and Schaap, 2018; Olschewski et al., 2018).

Edge-locked forests cannot grow without the necessary environment in which they exist, and soil is a crucial site of energy and material exchange in the ecosystem (Chai, 2018). Soil serves as the fundamental material for plant survival. It can provide conditions such as nutrition and environment needed for plant growth (Hansjürgens et al., 2018; Xu et al., 2013). In addition to being critical for plant growth, soil C, N, and P are essential markers of soil nutrient fertility (Cheng and Guo, 2024). Furthermore, their stoichiometric relationships generate valuable insights into biogeochemical cycling and equilibrium mechanisms, which are vital for assessing soil quality and recognizing the coupling relationship between soil nutrients (Chen Q. et al., 2022; Zhang et al., 2019). Relevant research has demonstrated that forest and grass vegetation types and densities should be rationally configured while developing a windbreak, sand-fixing desert-locked forest, and grass belt. Several configurations of plantation forests significantly impact soil C, N, and P and their ecological stoichiometry. Yu et al. (2024) demonstrated that pure forests of mountain poplar (*Populus davidiana*) should be prioritized in vegetation restoration in Luoshan, Ningxia. Yu et al. (2022) examined five degraded vegetation communities in Karst and demonstrated the critical role that N supply plays in restoring degraded forest vegetation. Furthermore, soil C, N, and P stoichiometric characteristics were appreciably affected by soil horizons. Typically, surface soils have higher C content and lower C/N/P ratios, whereas the opposite is true for deeper soils (Dong et al., 2024). For plantation forests in the western region of Guangdong Province, Wu et al. (2024) described the vertical distribution of soil carbon (C), nitrogen (N), and phosphorus (P) nutrients. They also revealed that soil horizons substantially influenced the stoichiometric characteristics of soil C, N, and P. In their study of central Himalayan vegetation, Joshi and Garkoti (2023) showed that SOC, TN, and TP content decreased with increasing soil depth. Although there are similarities between the existing related studies, no consistent conclusions have been drawn, indicating that there are still differences in the ecological stoichiometric characteristics of soil C, N, and P in various configuration types and soil depths at the regional scale.

Soil particle size is one of the most crucial physical properties of soil (Bayat et al., 2017). It is strongly correlated with the nutrients found in soil (Yuan et al., 2017), and variations in the coarseness and fineness of its particles directly affect soil nutrient changes (Nkheloane et al., 2012). Furthermore, soil particles are important indicators for classifying soil desertification (Bogunovic et al., 2017). Average particle size, sorting coefficient, kurtosis, skewness, and fractal dimension have emerged as crucial markers to assess soil particle changes in studies about the properties of soil particle size distribution (Pan et al., 2020). In an investigation of different types of *Populus alba*, Li et al. (2024) revealed that *Populus alba* considerably increased soil nutrients and fine particulate matter. Li X. L. et al. (2022) examined the variation characteristics of the fractal dimension of soil particles in red sand scrub sandpiles. They discovered that the fractal dimension had the same trend as fine particles, such as soil nutrients and clay meal particles. Gui et al. (2010) found a relationship between soil particle size distribution and elevation, plant community, soil pH, and moisture. Based on the previous research on the relationship between soil nutrients and soil particle size characteristics, this study aims to provide a reference for in-depth investigations into the composition of soil particles, the fractal dimension characteristics of these particles, and their interrelationships with C, N, P, and stoichiometric properties in the understory of edge-locked forests.

The Kubuqi Desert is the seventh-largest in China. The sand control measures of “locking in the north, blocking in the south, and cutting in the middle” have been used because of the topographical characteristics of the Kubuqi Desert. The Kubuqi Desert is 400 kilometers long from east to west, with an average width of 50 kilometers from north to south, covering a total area of more than 18,000 square kilometers and flowing southeastward as a whole due to the impact of more northwesterly winds. The “northern lock” is in the Kubuqi Desert north of the wind mouth place, leading to the formation of edge-locked forests. This forest belt tightly locates the desert’s edge, blocks the wind, and reduces the source of sand, improving the soil structure. Due to its unique geographic location, which is affected by rainfall and climate, the vegetation type of the edge-locked forest belt is diverse. The Desert Edge-locked Forests Project has become one of the elements of the typical management model. In this paper, six configurations of protection forests (*Salix psammophila*, *Elaeagnus angustifolia*, *Salix matsudana*, *Corethrodendron fruticosum + Salix psammophila*, *Caragana korshinskii + Populus simonii*, and *Elaeagnus angustifolia + Salix matsudana*) within the edge-locked forests in Kubuqi Desert were used as research objects. Systematic and in-depth analysis of the vertical differentiation of soil C, N, P, and ecological stoichiometric ratio and soil particle size characteristics of the 0–100-cm soil layer under protection forests of different configuration modes, this study’s conclusions can provide a scientific foundation for the biogeochemical cycle of soil C, N, and P in the Kubuqi Desert edge-locked forests. Meanwhile, the findings provide a scientific reference for the desert area’s vegetation restoration and improvement of the ecological environment. They also present a guide for constructing the local edge-locked forest vegetation. At the same time, the results can serve as a scientific reference for restoring and improving the ecological environment in desert regions. In addition, they provide advice on how to maintain local vegetation in edge-locked forests.

## 2 Materials and methods

### 2.1 Study area

**Figure 1:**
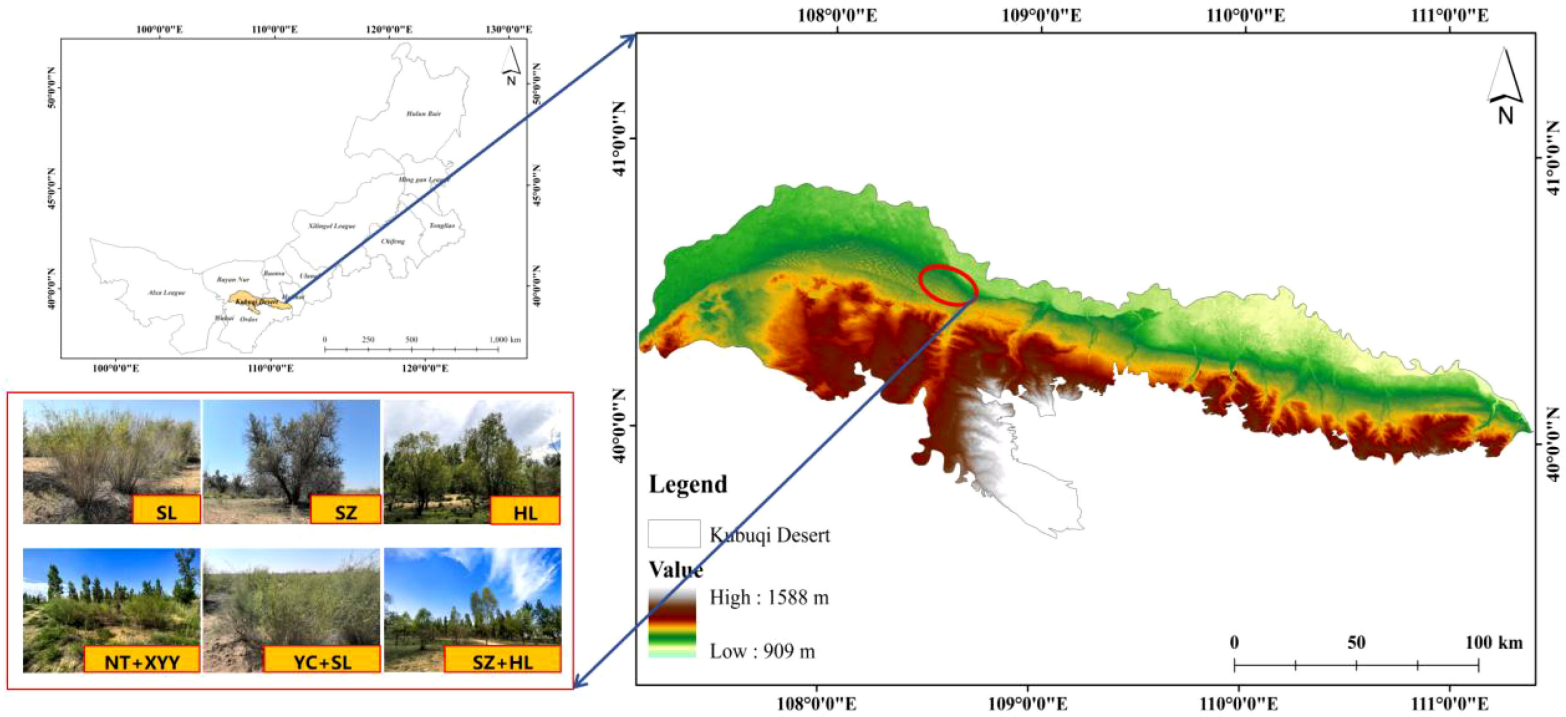
Location of the study region and sampling sites.

The northern edge of the Kubuqi Desert, located in Ordos City, Inner Mongolia Autonomous Region, was selected as the study area (**Figure 1**). The region is semi-arid and has a temperate continental monsoon climate. Wind and sand activities prevail from March to May. The average yearly temperature fluctuates between 5°C and 8°C with an average annual precipitation of 144–366 mm. The geomorphology type is dominated by fixed sandy land with flowing and semi-flowing dunes. The central tree species include *Salix psammophila*, *Elaeagnus angustifolia*, *Caragana korshinskii*, *Salix matsudana*, *Corethrodendron fruticosum*, *Populus simonii*, and other native species. The understory vegetation is primarily *Artemisia desertorum*, *Salsola collina*, *Psammochloa villosa*, and *Elymus dahuricus*.

### 2.2 Sample collection

Six different configurations (*Salix psammophila*, *Elaeagnus angustifolia*, *Salix matsudana*, *Corethrodendron fruticosum + Salix psammophila*, *Caragana korshinskii + Populus simonii*, and *Elaeagnus angustifolia + Salix matsudana*) of protection forest sample plots were selected in the study area. Considering the uneven distribution of herbaceous plants in the sample plots, three sample squares of 30 m × 30 m were established in every sample plot, and five soil samples were taken at equal distances along the diagonal of each sample square and mixed as one representative soil sample. The soil samples were undertaken using a soil auger at various depths: 0–10 cm, 10–20 cm, 20–30 cm, 30–40 cm, 40–50 cm, 50–60 cm, 60–70 cm, 70–80 cm, 80–90 cm, and 90–100 cm. Soil samples from each depth were mixed and placed in self-sealing bags, resulting in 180 soil samples (6 × 3 × 10). The gathered samples were then separated into two sections: one for determining soil nutrients and the other for analyzing soil particle size.

### 2.3 Determination of soil parameters

The soil particle size was measured using a laser particle sizer (Mastersize 2000; Malvern Instruments Ltd.) ranging from 0.01 *µ*m to 2000 *µ*m. The soil particle size findings were classified according to the United States Department of Agriculture (USDA) grading criteria, defining particles as clay (0–2 *µ*m), silt (2–50 *µ*m), and sand (50–2000 *µ*m).

Soil samples were dried naturally before being sieved through a 0.149-mm mesh. The potassium dichromate method was employed to ascertain the soil SOC content, the TN was assessed with an automatic Kjeldahl nitrogen tester, and the molybdenum blue colorimetric method was used to measure TP (Bao, 2000).

### 2.4 Calculating the parameters for soil particle size

The Folk-Ward graphical approach was applied to calculate particle size parameters, such as mean particle size (*d*_0_), standard deviation (*σ*_0_), skewness (*SK*), and kurtosis (*Kg*) (Folk and Ward, 1957). The calculation formula is expressed as follows:


$$\Phi =-log_{2}\text{D}$$

$$d_{0}=\frac{1}{3}(\Phi_{16}+\Phi_{50}+\Phi_{84})$$

$$\sigma_{0}=\frac{\left(\Phi_{84}-\Phi_{16}\right)}{4}+\frac{\left(\Phi_{95}-\Phi_{5}\right)}{6.6}$$

$$\text{SK}=\frac{\Phi_{16}+\Phi_{84}-2\Phi_{50}}{2(\Phi_{84}-\Phi_{16})}+\frac{\Phi_{5}+\Phi_{95}-2\Phi_{50}}{2(\Phi_{95}-\Phi_{5})}$$

$${\text{K}}_{\text{g}}=\frac{\Phi_{95}-\Phi_{5}}{2.44(\Phi_{75}-\Phi_{25})}$$

where *D* is the particle diameter, and $\Phi_{x}$ is the particle diameter corresponding to a cumulative volume fraction of *X*%.

### 2.5 Parameters for soil fractal dimension (D) computation

Utilizing the method of Tyler and Wheatcraft (1989), the particle size volume distribution characterizes the soil fractal model, which is used to calculate fractal dimensions.


$$\frac{{R_{i}}^{3-D}}{R_{\text{max}}}=\frac{V(\text{r}<R_{\text{i}})}{V_{T}}$$

where *R_i_* denotes the measured soil particle size; *R*_max_ is the maximum particle size; *V(r<R_i_*) is the soil’s volume, which is smaller than the measured particle size; *V_T_* is the sum of the volumes of the soil at each particle level; and *D* denotes the soil fractal dimension.

### 2.6 Data analysis

Microsoft Excel 2019 and SPPS 27.0 were used to organize the experimental data. Significant differences between soil nutrients and stoichiometric ratios under different configurations were analyzed via one-way ANOVA using Duncan’s method for multiple comparisons. The LSD method was applied for multiple comparisons, with the significance level of the differences being *α* = 0.05. R version 3.6.3 software “gplot2” package was applied to conduct the principal component analysis of soil factors under different configurations of edge-locked forests. The “stats” package was used to correlate soil C, N, and P contents and ecological stoichiometric ratios with particle size characteristics under different configurations of edge-locked forests. It was also used to draw correlation heat maps. Plotting was conducted using Origin 2021 software. The significance interval was defined as 95% level, and the graphs were reported as mean ± standard error.

## 3 Results

### 3.1 Characteristics of soil C, N, and P and their stoichiometry under different configurations of edge-locked forests

**Figure 2:**
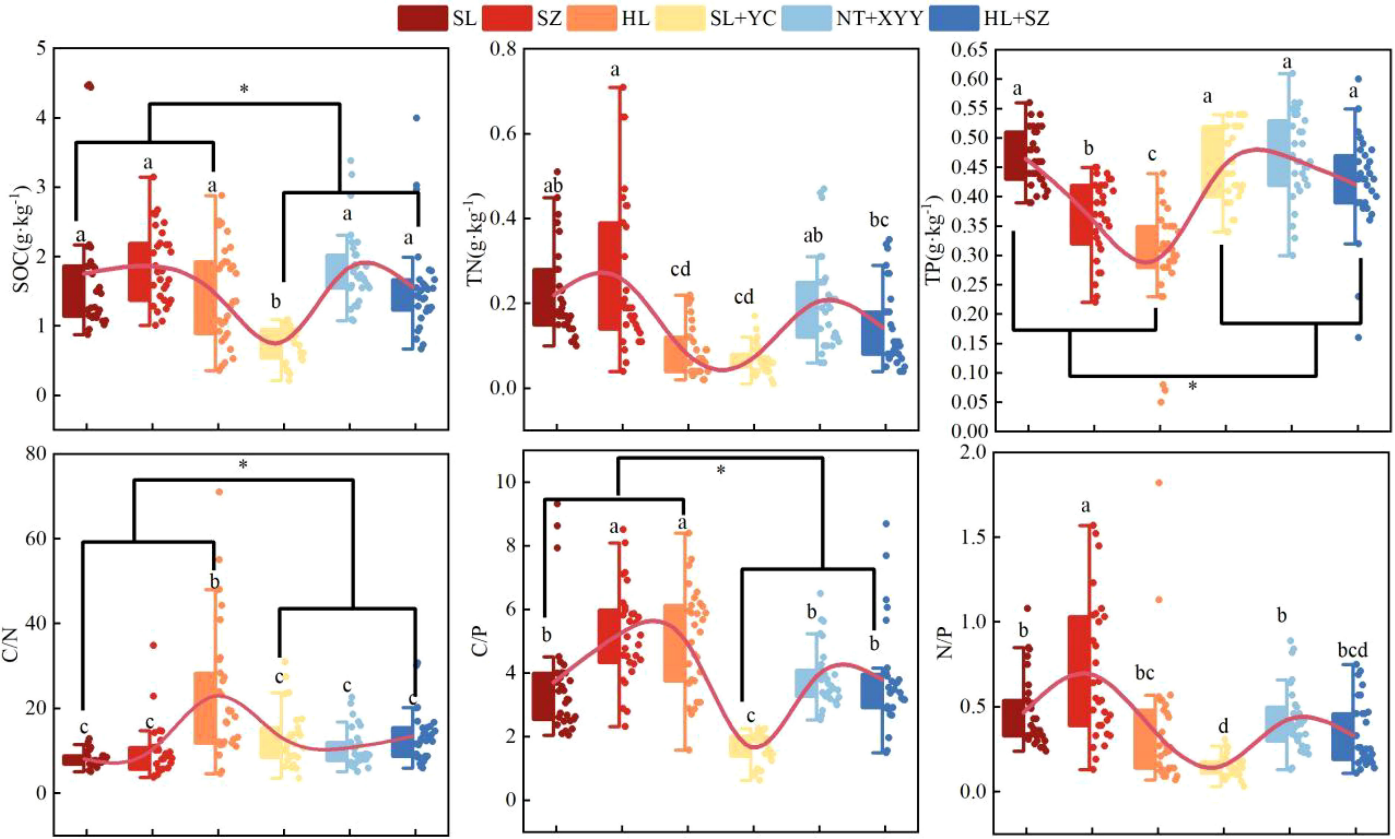
Changes in soil C, N, and P and their stoichiometric ratios among different configuration types. Different letters indicate significant differences (*P*<0.05) in soil C, N, and P between different configuration types of lock-edge forests.

From the distribution of soil SOC, TN, TP and stoichiometric characterization levels under different configurations of edge-locked forests (**Figure 2**), soil SOC, TN, TP, C/N, C/P, and N/P in the study area were significantly different under different configuration types of edge-locked forests (P<0.05). SOC and TN were considerably lower among the six configuration types. In contrast, SZ was significantly higher (*P*<0.05) than others. Mixed forests (SL + YC, NT + XXY, HL + SZ), as well as SL, had higher TP (*P*<0.05) than the other forest allocations. Overall, SOC was significantly higher (*P*<0.05) under monocultures (SL, SZ, and HL) than under mixed forests (SL + YC, NT + XXY, HL + SZ). The higher SOC also caused significantly higher (*P*<0.05) C/N and C/P under monocultures than in mixed forests. TN did not exhibit significant differences, and TP was considerably higher (*P*<0.05) in mixed forests than in monocultures.

**Figure 3:**
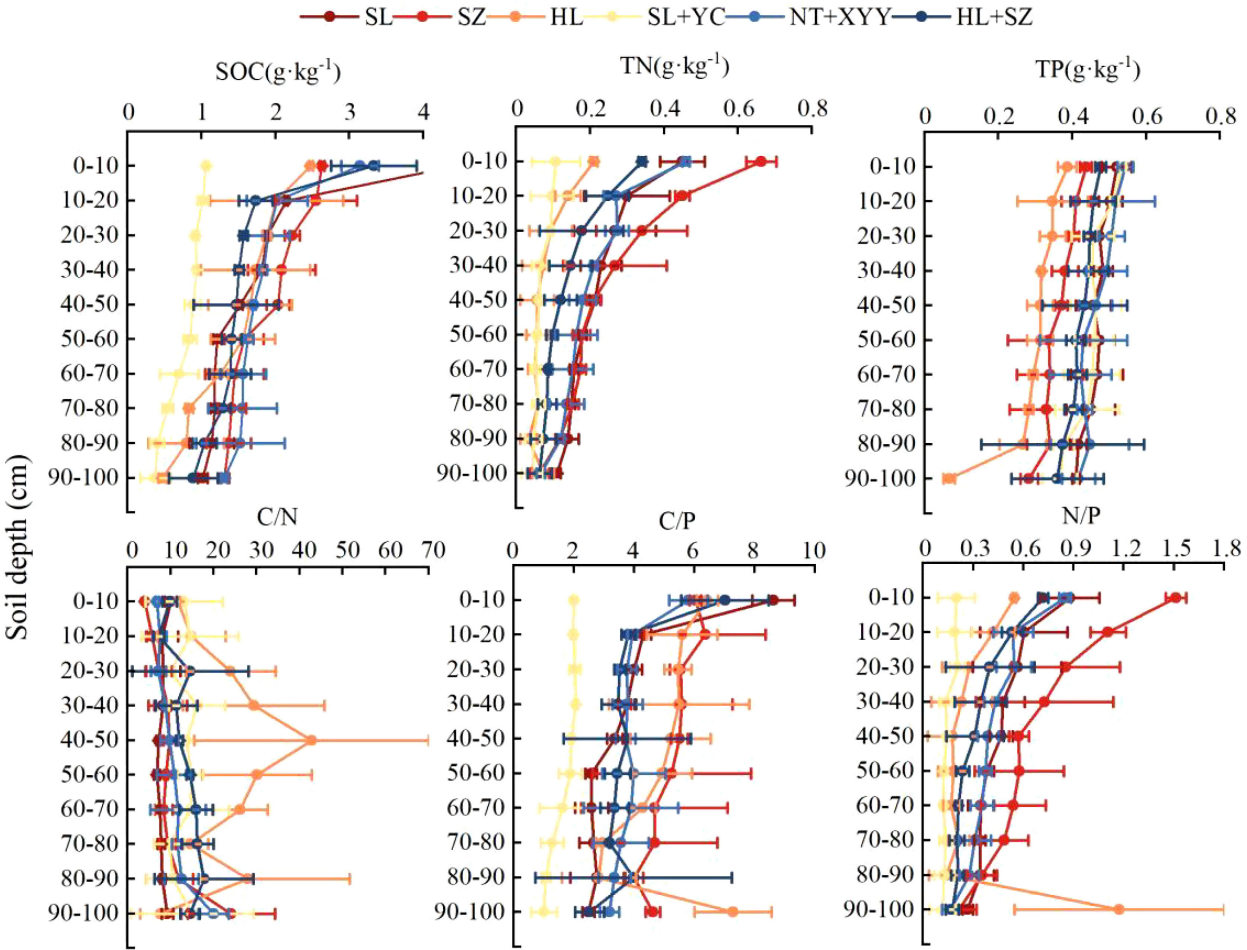
Distribution of soil C, N, and P and their stoichiometric ratios with soil depths in different configurations.

From the vertical distribution of soil SOC, TN, TP, and stoichiometric characteristics under different configurations of edge-locked forests (**Figure 3**), soil SOC, TN, TP, C/N, C/P, N/P, C/N, and N/P of the 0–100-cm soil layer under the six different configurations of edge-locked forests were all substantially different among different soil layers (*P*<0.05). Furthermore, the overall content of SOC, TN, and TP gradually declined with increasing soil depth. However, the magnitude of the TP change was not evident. Soil SOC, TN, TP, and their stoichiometric ratios under different configurations demonstrated epimerization, where the nutrient content in the 0–20 cm surface soil was higher than that in the depth of 20–100 cm. In contrast, the epimerization of C/N was not as pronounced as that of C/P and N/P. Meanwhile, C/P and N/P under the different configurations of edge-locked forests reduced with increasing soil depth. In contrast, C/N displayed an opposite trend.

### 3.2 Characterization of soil particle composition and parameters under different configurations of edge-locked forests

The horizontal distribution of soil particle size under different configurations of edge-locked forests (**Tables 1**, **2**) revealed that soil particle size composition in numerous edge-locked forest configurations was basically the same, which primarily comprised sand, with the content of sand being about 76.13~99.09%. This trend was followed by silt with 0.64~23.16% and clay with the lowest content of 0.10~2.6%. From the distribution of silt and clay, SL, SZ, NT + XYY, and SZ + HL were substantially more abundant than HL and YC + SL, with SL and NT + XYY having the most silt and clay.

**Table 1:** Basic information on different configuration types of edge-locked forests.

Type	Latitude (N°)	Longitude (E°)	Main vegetation	Vegetation cover	Site conditions
SL	40°61′	108°58′	*Artemisia ordosica*, *Phyllostachys propinqua*, *Grubovia dasyphylla*, *Incarvillea sinensis*, *Sophora alopecuroides*, *Agriophyllum pungens*	>50%	fixed sandy land
SZ	40°58′	108°61′	*Corispermum mongolicum*, *Cynanchum chinense*	>50%	
HL	40°58′	108°60′	*Corispermum mongolicum*, *Artemisia ordosica*, *Phragmites australis*, *Echinops gmelini*	>50%	
YC+SL	40°51′	108°58′	*Amorpha fruticosa*, *Artemisia ordosica*, *Corispermum hyssopifolium*, *Artemisia desertorum*, *Phyllostachys propinqua*	>45%	
NT+XYY	40°53′	108°64′	Setaria viridis, *Corispermum hyssopifolium*, *Artemisia desertorum*, *Salsola collina*	>65%	
SZ+HL	40°55′	108°63′	*Corispermum mongolicum*, *Echinops gmelini*, *Artemisia ordosica*	>40%	

SL is *Salix psammophila*, SZ is *Elaeagnus angustifolia*, HL is *Salix matsudana*, YC + *SL is Corethrodendron fruticosum+Salix psammophila*, NT + XYY is *Caragana korshinskii+Populus simonii*, and SZ + HL is *Elaeagnus angustifolia* + *Salix matsudana*.

According to the vertical distribution of soil particle size under different configurations of edge-locked forests (**Table 2**), the vertical distribution patterns of soil particle size composition within the 0–100 cm soil layer varied among different configurations of edge-locked forests. These patterns can generally be categorized into three types. (1) As the soil layer becomes deeper, the content of clay and silt particles generally shows a change in decreasing, then increasing, and decreasing. Represented by SL and SZ + HL, clay and silt contents were significantly higher in the 50-70-cm layer than in all other layers, and the 20-30-cm layer has the lowest contents (*P*<0.05). (2) There is no significant change in the grain levels with increasing soil layer depth. HL and YC + SL are represented. (3) As the soil layer becomes deeper, the content of coarse sand gradually increases, represented by SZ and NT + XYY.

**Table 2:** Characteristics of soil particle size distribution under different configurations of lock-edge forests.

Type		SL	SZ	HL	YC+SL	NT + XYY	SZ + HL
Soil Depth (cm)	Soil Particle Size (%)						
0–10	Clay Silt Sand	0.37 ± 0.07 b 13.20 ± 1.71 b 86.42 ± 1.74d	0.27 ± 0.07 c 7.62 ± 0.93 c 92.12 ± 1.00 c	0.17 ± 0.05 d 2.00 ± 0.21 de 97.83 ± 0.24 ab	0.14 ± 0.04 d 1.22 ± 0.18 e 98.64 ± 0.22 a	0.61 ± 0.12 a 23.26 ± 2.68 a 76.13 ± 2.75 e	0.20 ± 0.04 cd 6.98 ± 0.55 c 92.82 ± 0.57 c
10–20	Clay Silt Sand	0.29 ± 0.08 c 4.89 ± 0.62 c 94.81 ± 0.7 d	0.39 ± 0.07 b 7.08 ± 0.49 b 92.54 ± 0.55 e	0.14 ± 0.05 d 1.19 ± 0.28 e 98.67 ± 0.33 ab	0.11 ± 0.04 d 0.80 ± 0.12 e 99.09 ± 0.16 a	0.58 ± 0.13 a 17.47 ± 1.14 a 81.95 ± 1.24 f	0.16 ± 0.05 d 2.43 ± 0.57 d 97.41 ± 0.61 b
20–30	Clay Silt Sand	0.31 ± 0.05 b 3.25 ± 0.28 c 96.43 ± 0.32 c	0.26 ± 0.07 bc 4.00 ± 0.58 b 95.73 ± 0.57 d	0.12 ± 0.04 e 0.77 ± 0.18 f 99.11 ± 0.22 a	0.13 ± 0.04 e 0.85 ± 0.16 f 99.02 ± 0.2 a	0.59 ± 0.09 a 15.04 ± 0.96 a 84.37 ± 1.01 e	0.16 ± 0.04 de 1.65 ± 0.22 e 98.19 ± 0.26 c
30–40	Clay Silt Sand	0.51 ± 0.08 a 5.25 ± 0.65 c 94.24 ± 0.71 c	0.43 ± 0.08 a 7.07 ± 1.19 b 92.5 ± 1.24 d	0.19 ± 0.05 a 1.07 ± 0.16 e 98.74 ± 0.20 a	0.21 ± 0.07 a 1.09 ± 0.19 e 98.69 ± 0.25 a	0.51 ± 0.08 a 12.73 ± 0.87 a 86.76 ± 0.9 e	0.35 ± 0.07 b 3.35 ± 0.51 d 96.3 ± 0.57 b
40–50	Clay Silt Sand	1.68 ± 0.32 a 19.55 ± 2.3 a 78.77 ± 2.53 e	0.50 ± 0.09 b 7.22 ± 0.48 c 92.28 ± 0.52 c	0.15 ± 0.03 e 0.80 ± 0.09 e 99.05 ± 0.12 a	0.33 ± 0.07 cd 1.50 ± 0.20 e 98.17 ± 0.26 ab	0.47 ± 0.07 b 7.68 ± 0.60 c 91.85 ± 0.66 c	0.44 ± 0.08 bc 9.47 ± 1.97 b 90.09 ± 2.00 d
50–60	Clay Silt Sand	2.6 ± 0.50 a 22.34 ± 2.25 a 75.06 ± 2.60 e	0.45 ± 0.10 b 5.66 ± 0.98 b 93.88 ± 1.06 d	0.10 ± 0.03 cd 0.64 ± 0.09 e 99.27 ± 0.12 a	0.19 ± 0.05 cd 1.09 ± 0.14 e 98.72 ± 0.19 a	0.37 ± 0.05 bc 3.73 ± 0.35 c 95.9 ± 0.39 c	0.30 ± 0.08 bcd 6.16 ± 2.08 b 93.54 ± 2.15 d
60–70	Clay Silt Sand	1.67 ± 0.24 a 18.99 ± 2.21 a 79.34 ± 2.41d	0.42 ± 0.12 b 3.82 ± 0.77 c 95.76 ± 0.89 b	0.24 ± 0.05 c 1.50 ± 0.19 d 98.26 ± 0.24 a	0.20 ± 0.05 c 1.10 ± 0.14 d 98.7 ± 0.19 a	0.41 ± 0.07 b 3.81 ± 0.33 c 95.78 ± 0.39 b	0.47 ± 0.09 b 8.31 ± 1.83 b 91.22 ± 1.90 c
70–80	Clay Silt Sand	1.05 ± 0.41 a 14.94 ± 6.69 a 84.01 ± 7.08 a	0.42 ± 0.11 b 3.06 ± 0.49 bc 96.53 ± 0.59 a	0.24 ± 0.06 cd 1.57 ± 0.25 bc 98.2 ± 0.30 ab	0.15 ± 0.07 d 1.00 ± 0.28 c 98.86 ± 0.34 a	0.45 ± 0.10 b 3.62 ± 0.87 bc 95.93 ± 0.95 b	0.33 ± 0.07 bc 3.17 ± 0.43 bc 96.50 ± 0.50 ab
80–90	Clay Silt Sand	1.01 ± 0.26 a 14.07 ± 3.34 a 84.91 ± 3.53 c	0.37 ± 0.07 b 2.46 ± 0.29 b 97.17 ± 0.35 b	0.16 ± 0.04 d 1.03 ± 0.16 c 98.81 ± 0.20 a	0.18 ± 0.05 d 1.08 ± 0.15 c 98.74 ± 0.19 a	0.28 ± 0.10 cd 1.96 ± 0.39 bc 97.77 ± 0.49ab	0.27 ± 0.07 cd 2.17 ± 0.32 bc 97.56 ± 0.38 ab
90–100	Clay Silt Sand	0.45 ± 0.13 b 11.71 ± 1.52 a 87.84 ± 1.64 e	0.59 ± 0.08 a 3.28 ± 0.25 c 96.13 ± 0.32 c	0.20 ± 0.05 d 1.06 ± 0.15 e 98.73 ± 0.20 a	0.20 ± 0.05 d 1.22 ± 0.16 e 98.57 ± 0.21 a	0.37 ± 0.08 c 2.33 ± 0.30 d 97.3 ± 0.38 b	0.66 ± 0.10 a 4.15 ± 0.40 b 95.19 ± 0.50 d

The average ± standard error is shown above. Different letters indicate significant (*P*<0.05) differences in grain size composition between different configuration types of edge-locked forests.

**Figure 4:**
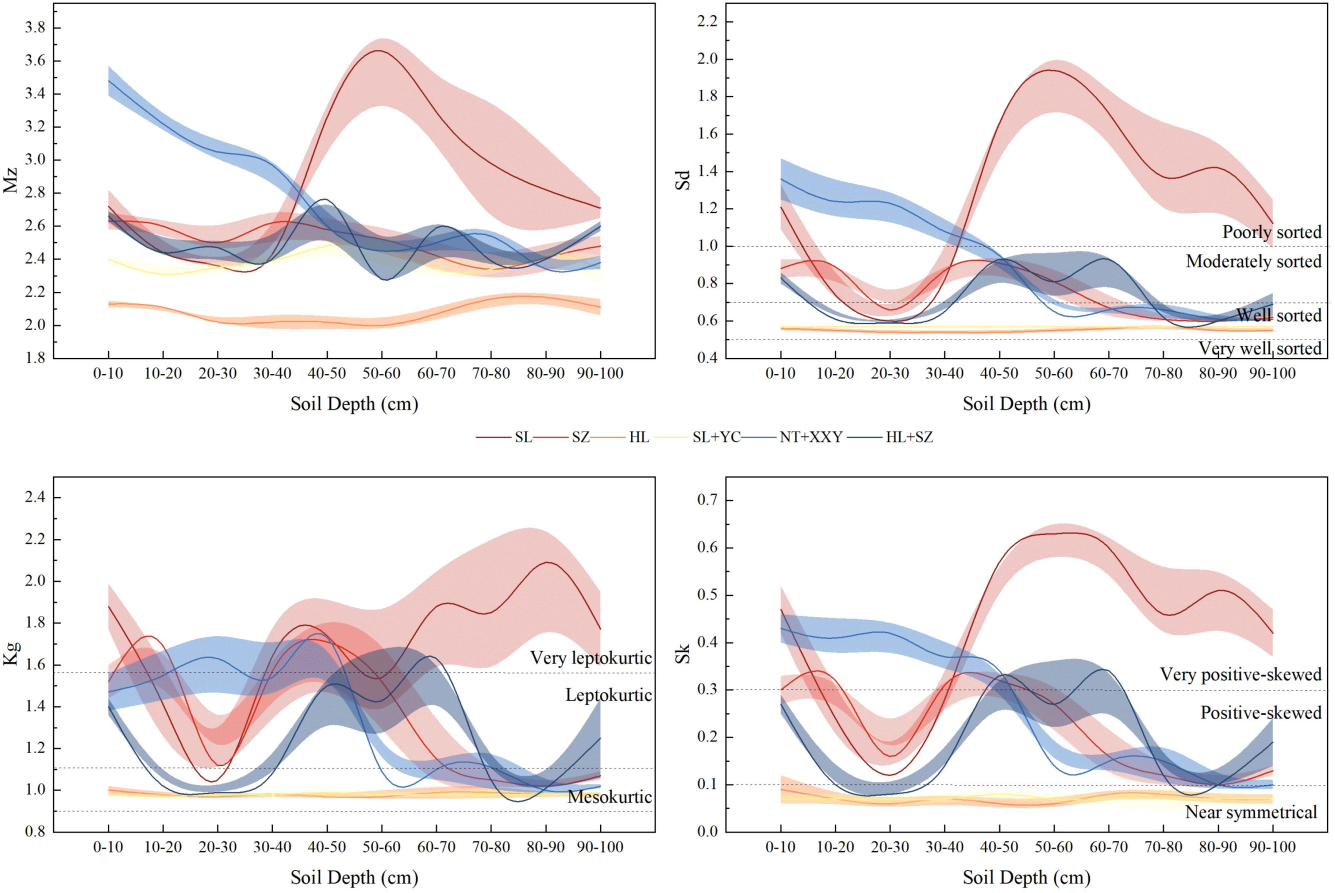
Characteristics of soil particle size parameters between different configuration types.

From the characteristic-level distribution of soil particle size parameters under different configurations of edge-locked forests (**Figure 4**), the average soil *M_Z_* under the six configurations of edge-locked forests, SL, SZ, HL, YC + SL, NT + XYY, and SZ + HL, were 2.87, 2.51, 2.08, 2.38, 2.75, and 2.50, respectively. SL has a finer texture, and NT + XYY is second. The average *Sd* of the soil under the six configurations of SL, SZ, HL, YC + SL, NT + XYY, and SZ + HL in the edge-locked forests was 1.26, 0.75, 0.55, 0.57, 0.91, and 0.73, respectively. SL was poorly sorted, SZ, NT + XYY, and SZ + HL were moderately sorted, and HL and YC + SL were well sorted. The average soil *Kg* values under the six configurations of SL, SZ, HL, YC + SL, NT + XYY, and SZ + HL edge-locked forests were 1.69, 1.34, 0.98, 0.98, 1.32, and 1.24, respectively. HL and YC + SL were moderate, SZ, NT + XYY, and SZ + HL were sharp and narrow, and SL exhibited very sharp and narrow. The average soil *SK* values under the six configurations of SL, SZ, HL, YC + SL, NT + XYY, and SZ + HL in the edge-locked forests were 0.43, 0.22, 0.07, 0.07, 0.26, and 0.19, respectively. HL and YC + SL were nearly symmetrical, HL, NT + XYY, and SZ + HL showed positive bias, and SL demonstrated very positive bias.

From the vertical distribution of soil particle size parameter characteristics under the different configurations of edge-locked forests (**Figure 4**), the average particle size and fractal dimension displayed the same change trend with an increase in soil depth. The overall sortability of the soil profiles was moderately favorable, with nearly symmetrical positive skewness and moderately narrow kurtosis, except for SL. Notably, the HL, SL + YC skewness and kurtosis did not vary significantly (*p*>0.05) between the soil layers.

The characteristic-level distribution of the fractal dimension of soil particle size under the different configurations of edge-locked forests (**Table 3**), except for SL, reveals that the fractal dimension was highest in the NT + XYY configuration type and lowest in the HL and YC + SZ configuration types. It was significantly different (*p*<0.05) from the other configurations. This outcome indicates that NT + XYY has improved dramatically the soil structure and particle size distribution. Soil particle size fractal dimension varied on the vertical gradient in different configuration types of edge-locked forests but also displayed a certain pattern. The soil fractal dimension decreased with increasing soil depth in NT+XYY edge-locked forests, while all other configurations of edge-locked forests showed a general trend of decreasing, then increasing and then decreasing with increasing soil depth, with a minimum in the 20-30 cm soil layer, increasing to a maximum and then decreasing around the 40-50 cm soil layer, and then increasing to the 90-100 cm soil layer.

**Table 3:** Characteristics of soil fractal dimension distribution under different configurations of edge-locked forests.

Type		SL	SZ	HL	YC + SL	NT + XYY	SZ + HL
Soil Depth (cm)	D						
0-10		2.11 ± 0.03 d	2.03 ± 0.05 cd	1.88 ± 0.05 ab	1.83 ± 0.06 bcd	2.22 ± 0.03 a	1.99 ± 0.03 bc
10-20		2.01 ± 0.05 e	2.08 ± 0.03 ab	1.82 ± 0.06 c	1.78 ± 0.06 d	2.19 ± 0.04 ab	1.89 ± 0.07 d
20-30		2.00 ± 0.03 e	1.99 ± 0.04 cd	1.77 ± 0.06 d	1.80 ± 0.06 d	2.19 ± 0.02 ab	1.88 ± 0.04 d
30-40		2.10 ± 0.03 d	2.10 ± 0.03 a	1.86 ± 0.05 bc	1.89 ± 0.05 b	2.16 ± 0.03 b	2.02 ± 0.04 b
40-50		2.34 ± 0.03 b	2.12 ± 0.03 a	1.81 ± 0.04 c	1.97 ± 0.04 a	2.11 ± 0.02 c	2.12 ± 0.03 a
50-60		2.41 ± 0.03 a	2.09 ± 0.04 ab	1.73 ± 0.06 d	1.88 ± 0.04 b	2.04 ± 0.02 de	2.03 ± 0.06 b
60-70		2.34 ± 0.03 b	2.05 ± 0.07 bc	1.91 ± 0.04 a	1.88 ± 0.04 b	2.06 ± 0.03 d	2.11 ± 0.04 a
70-80		2.25 ± 0.08 c	2.04 ± 0.05 c	1.92 ± 0.05 a	1.81 ± 0.12 cd	2.06 ± 0.05 d	2.01 ± 0.04 b
80-90		2.25 ± 0.05 c	2.01 ± 0.03 cd	1.84 ± 0.05 bc	1.87 ± 0.05 bc	1.95 ± 0.07 f	1.96 ± 0.05 c
90-100		2.13 ± 0.05 d	2.10 ± 0.02 a	1.87 ± 0.04 ab	1.89 ± 0.04 b	2.01 ± 0.04 e	2.13 ± 0.03 a

The average ± standard error is shown above; different letters indicate that *D* differs significantly (*P*<0.05) between different configuration types of edge-locked forests.

### 3.3 Relationships between soil C, N, and P contents and ecological stoichiometric ratios and particle size characteristics in different configurations of edge-locked forests

**Figure 5:**
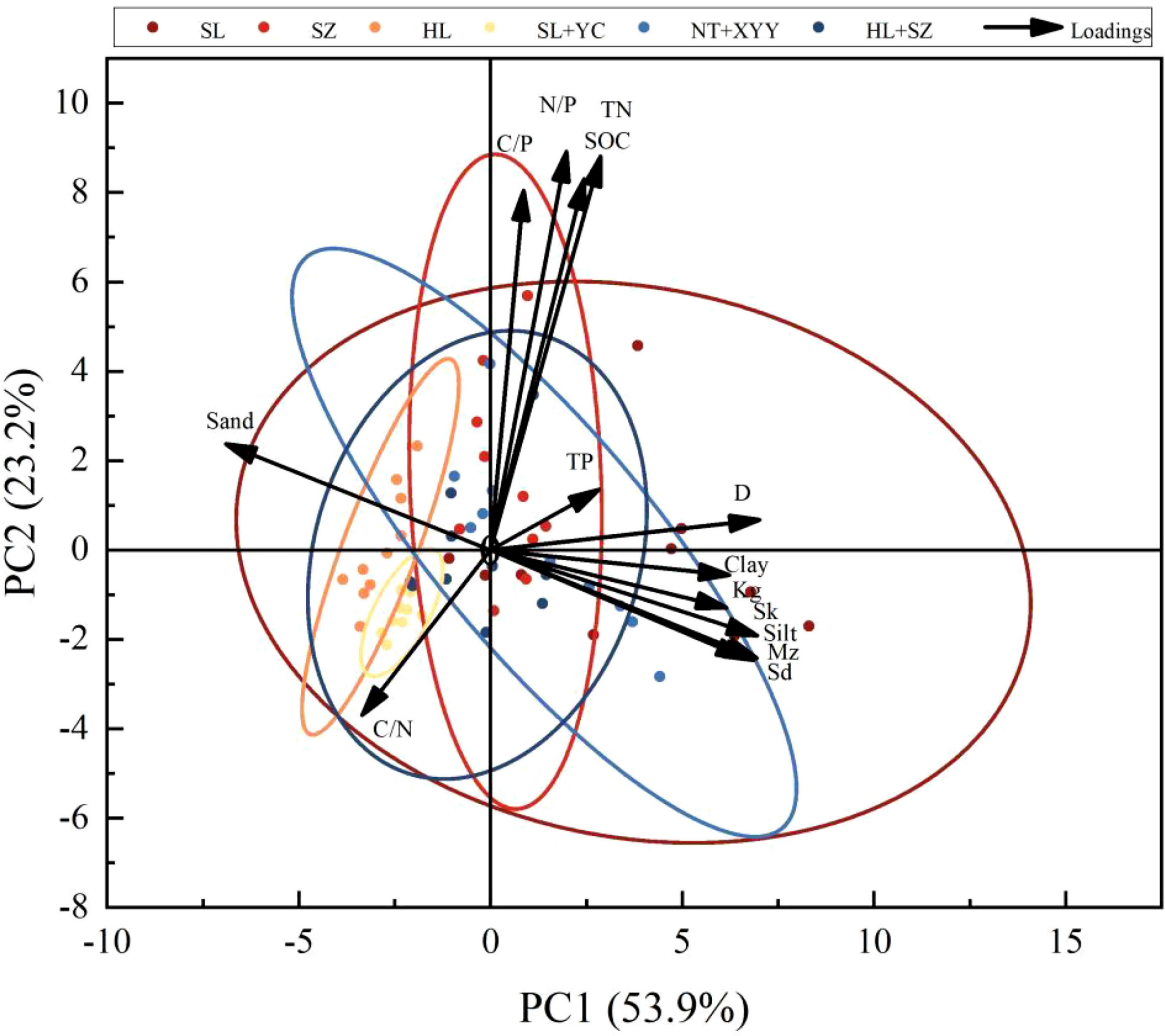
Principal component analysis of soil properties among different configuration types.

In the principal component analysis, the 95% confidence interval distribution demonstrated differences in the effects of different configurations of edge-locked forests on soil indicators (**Figure 5**), with significant differences between SL and NT + XYY and the other groups in the six groups and insignificant differences and similarities between HL, SL + YC, and HL + SZ stands. The first and second principal components explained 53.9% and 23.2%, respectively, which explained a total of 77.1% of the variation pattern of soil factors, and could comprehensively reflect the dominant factors affecting the status of each soil indicator. The largest contributions of PC1 were Sd, Mz, silt, and D, indicating that PC1 was primarily related to Sd, Mz, silt, and D of different configurations of edge-locked forest soils. N/P, SOC, and TN were the largest contributors to PC2, revealing that PC2 was primarily related to N/P, SOC, and TN of different configurations of edge-locked forest soils. The different configurations of edge-locked forests overlapped on PC1 and PC2; therefore, the soil conditions of the six different configurations of edge-locked forests could not be completely differentiated by PC1 and PC2, and the various properties of the soils were closely related.

**Figure 6:**
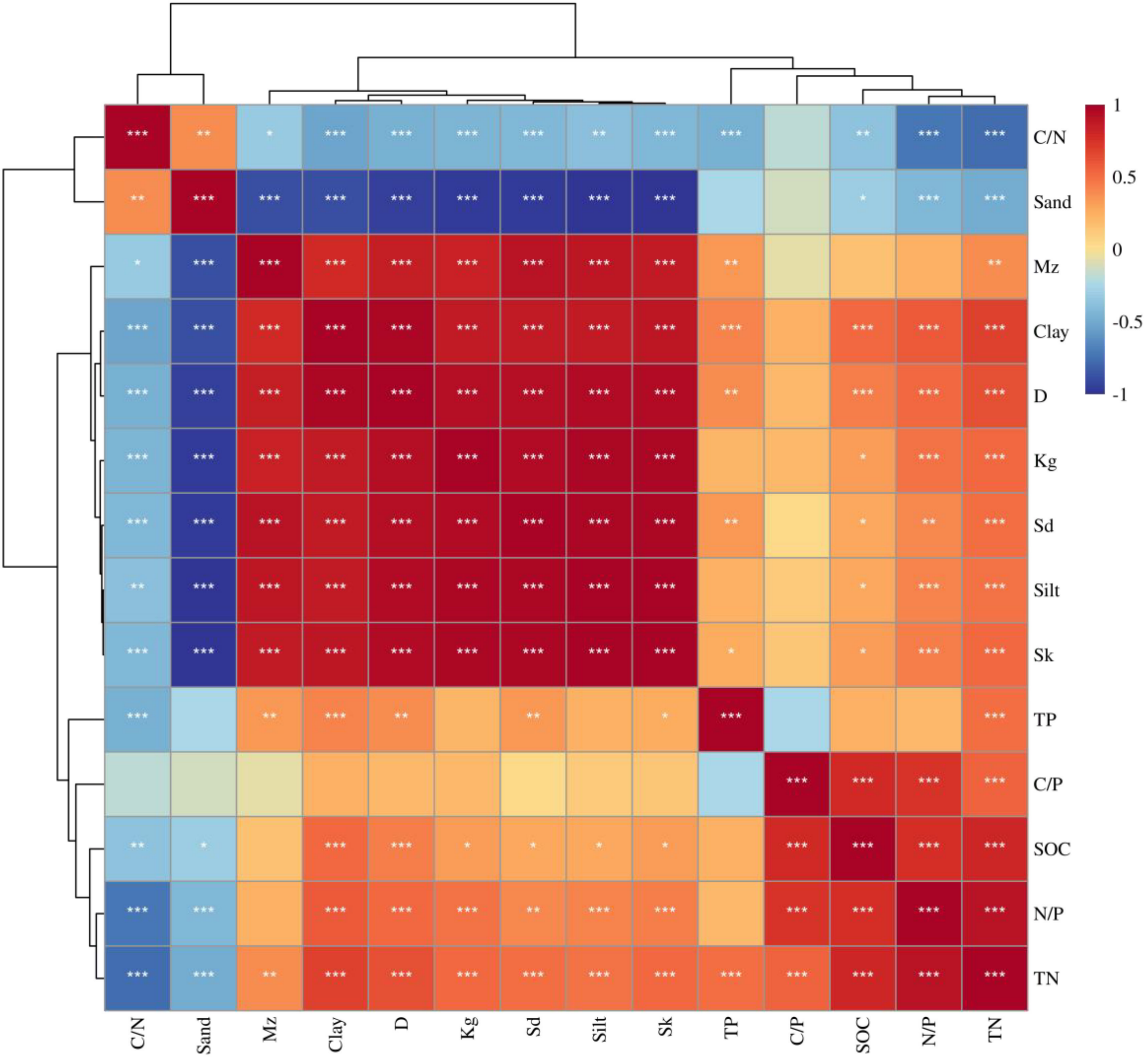
Heat map of Pearson correlation between soil properties among different configuration types. (At the 0.05, 0.01, and 0.001 levels, respectively, significant correlation is indicated by the symbols *, **, and ***).

Correlations and asterisks respond to significance via color changes in the heat map. Pearson’s correlation analysis demonstrated (**Figures 5**, **6**) that soil C, N, and P content and ecological stoichiometric ratios differed in correlation degree between different configuration types and particle size characteristics. In addition, SOC positively correlated with Sk, Silt, *Sd*, and *Kg* (*P*<0.05) while demonstrating a negative connection with sand and C/N. Soil TN was highly associated with all indicators. Soil TP exhibited a highly significant positive correlation with TN and clay (*P*<0.001). In addition, TP presented a significant positive correlation with *Sd*, D, and Mz (*P* < 0.01) while showing a highly significant negative correlation with the C/N (*P*<0.001). C/N was significantly and positively correlated with sand (*P*<0.01) and had no significant relationship with C/P. Furthermore, C/N exhibited a significant negative correlation with each of the other indices. C/P was significantly and positively correlated solely with SOC. In contrast, N/P and TN exhibited highly significant positive correlations (*P*<0.001). N/P showed a highly significant negative correlation with sand and C/N (*P*<0.001), no significant relationship with Mz and TP, and a significant positive correlation with all other indicators.

## 4 Discussion

### 4.1 Effects of different configurations of edge-locked forests on soil carbon, nitrogen, and phosphorus and their stoichiometric characteristics

Soil C, N, and P content and stoichiometric ratios were strongly impacted by vegetation type (Huang et al., 2024). The average soil C, N, and P contents of the six configuration types of edge-locked forests ranged from 0.77 to 1.86 g·kg^-1^, 0.07 to 0.26 g·kg^-1^, and 0.2 to 0.6 g·kg^-1^, respectively. According to the grading standard of the second soil nutrient census in China (National Soil Census Office, 1998), the soil C, N, and P contents were grade 4~5 (0.2~0.6 g·kg^-1^), grade 6 (<6.0 g·kg^-1^), and grade 6 (<0.5 g·kg^-1^), respectively. This result suggests that in the lock border forests in this study area, the soil was relatively deficient in C and N, the organic C sequestration capacity of the soil was low, and the P content of the soil was average. This phenomenon may be caused by the site being a fluvial sandy area with sandy and fragile soils, greatly affecting soil organic C sequestration. Meanwhile, the main component analysis results indicated that soil SOC and TN exerted the greatest influence on the PCA2 axis (**Figures 5**, **6**), suggesting that the soil in this research area is more likely to be limited by C and N elements.

In previous studies, soil C, N, and P soil nutrients are important components primarily affected by apoplastic decomposition, root secretions, soil mineralization, and microbial activities (Gao et al., 2014). There were differences in the C, N, and P contents of soils under six different configuration types of lock-edge forests in the Kubuqi Desert. The SOC and TN contents of SZ, NT + XYY, and SL soils were considerably higher than those of other configuration types. This trend is consistent with the results of Wang et al. (2022) and Li et al. (2022) in the transition zone along the Yellow River. This phenomenon is primarily attributed to differences in the quantity and quality of root secretions in different configuration types, as well as the varying rates of decomposition of apoplastic material in other vegetation, which causes differences in the impact on nutrients (Yang et al., 2021; Angst et al., 2017). At the same time, climate change and human activities can also lead to changes in the quality and quantity of apoplastic and root inputs, which in turn can affect below-ground biogeochemical processes in forest ecosystems (Abay et al., 2024). The root system of SL has strong sprouting power and a wide distribution range. The decomposition of its apoplastic material is relatively slow, and it gradually accumulates on the soil surface to create an organic layer and increase the organic C content of the soil. Its root secretion comprises N compounds, which can provide a nitrogen source for soil microorganisms and enhance the soil (Yang et al., 2018). SZ is also affected by plant apoplastic decomposition, root secretions, and larger biomass and more C fixed by photosynthesis compared to SL (Shi et al., 2022). In NT + XYY, NT rhizobacteria play a crucial role in nitrogen fixation, and the pairing of the two significantly affects soil improvement (Wen et al., 2024). Another essential factor is the abundance of plant species, high biomass, and high vegetation cover in SZ, NT + XYY, and SL (**Table 1**). Related studies have shown that increased herbaceous biomass and vegetation cover can enable soil C and N biological processes and surface soil microbial activity, leading to higher surface soil SOC and STN contents (Lu et al., 2023). The study findings demonstrated that the soil mixtures under the six configuration types of lock-edge forests were higher in total P than the single species, with NT + XYY being the most pronounced. Li et al. (2022) are consistent with this research’s results because the synergistic effect of mixed forests can more effectively convert insoluble P into effective P in the soil than a single tree species (Qin et al., 2024). In addition, the complementary distribution of root systems in mixed forests can fully use P at different levels in the soil and increase the soil’s total P content through root turnover and apoplastic return (Du et al., 1995).

The C, N, and P ratios of the soils under six different configuration types of lock-edge forests in the Kubuqi Desert showed variations. The average C/N, C/P, and N/P contents of the soils of the six configuration types of lock-edge forests ranged from 8.14 to 22.95 g·kg^-1^, 1.68 to 5.22 g·kg^-1^, and 0.15 to 0.69 g·kg^-1^. C/N fluctuated around the national average (11.90), and the C/P and N/P ranges were considerably lower than the national average (61 and 5.2) (Tian et al., 2010). It indicates that nitrogen is the restricting nutrient element for the growth of edge-locked forests in the region, but P is more effective. The C/N indicator primarily evaluates C and N cycling in the soil. When the mean C/N ratio is high, the decomposition rate of its organic matter is slow; conversely, when the mean C/N ratio is low, soil decomposition releases N pigments, which increase the N elemental content of the soil (Wang et al., 2020). HL, HL + SZ, and SL + YC soil C/N were higher than the average among the six configuration types, probably because of the slow decomposition of soil organic matter in these three edge-locked forests, limiting the rate of nutrient cycling. Although SL, SZ, NT + XYY soil C/N was lower than the mean value, these three configurations of lock-edge forest soil nitrogen transformation and accumulation efficiency were higher. Generally, vegetation growth is N-limited when N/P<10 (Tyler and Wheatcraft, 1989). Vegetation at this site may have been constrained by soil N during growth. Of the six types, SL + YC soil C/P and N/P were considerably lower than the other configurations, indicating that the soil P effectiveness of SL + YC forest was much higher than that of the different tree species. At the same time, the degree of limitation by N was stronger compared to the other five forest stands. This is also consistent with the Wu et al. (2024). Therefore, nitrogen fertilizers and exogenous nitrogen inputs should be rationally applied to improve nutrient limitation and increase soil fertility for ecological restoration of the area’s edge-locked forests.

The SOC and TN of the understory soil of the edge-locked forests at this site were associated with the C/P and N/P contents (**Figure 5**). The soil SOC and TN exhibited highly significant positive associations with both soil C:P and soil N:P, as well as with soil TN. This phenomenon is similar to the findings of Chen Y. et al. (2022) because of the synchronized response of C and N in the understory soil of this site to the same environmental factor, and there is a close coupling relationship (Zhang et al., 2024). It reveals that the soil C/N of the edge-locked forests at this site is relatively spatially stable, confirming the dynamic equilibrium theory of ecological stoichiometry.

### 4.2 Changes in soil C, N, and P and their stoichiometry with soil depth in different configurations of edge-locked forests

Soil depth significantly impacts soil C, N, and P levels and stoichiometric ratios (Li et al., 2021). In this research, the C and N contents of the soil under six different types of edge-locked forests in the Kubuqi Desert decreased with the increase of soil depth and exhibited the phenomenon of “surface aggregation.” The primary reason behind this was that the surface soil was subjected to the return of nutrients by the external environmental factors and the vegetation litter, as well as the aggregation of plant and animal microbial residues, which resulted in an accumulation of nutrients in the surface soil (Chapin et al., 2011; Logah et al., 2020). There was no discernible variation in P concentration with increasing depth. This trend agrees with the findings of Sun et al. (2023) in a previous investigation in Southwest China. Similar conclusions were obtained for related studies in the northern grasslands of China (Lu et al., 2023). Relative to soil C and N content, the P content did not vary much in different soil horizons, primarily because the slow biotransformation rate during the cycling of P. Microbial activities in the soil have less influence on P. The biological role of P in the soil is relatively small (Da Silva et al., 2022).

Soil C/P and N/P steadily dropped with the increase in soil depth, agreeing with the findings of Zhang et al. (2020). This outcome may be ascribed to the decrease of soil SOC and TN contents caused by soil microbial activity and the downward leaching migration of organic matter (Wang et al., 2021). The opposite pattern of C/N variation was consistent with Yang et al.’s vertical profile pattern of soil in Jinji’er forest in Maowusu Sandland (Yang and Liu, 2019). Except for HL, the differences in C/N among the various soil layers were insignificant, indicating that the C/N values were relatively stable, reflecting the synergistic response of C and N elements to changes in the external environment. This is similar to the findings of Ma et al.’s investigation in the Baiyu Mountain area (Ma et al., 2023).

### 4.3 Effect of different configurations of edge-locked forests on soil particle composition and parameter characteristics

The soil particle size was primarily sand under the different configurations of edge-locked forests. At the same time, the silt and clay contents of SL and NT + XYY were considerably higher than the other four configurations due to the depositional effect of the protective forests (Chen et al., 2020). It has been shown that silt and clay can be suspended over long distances by wind (Qin, 2009). The site is windy, and the silt and clay grain size is small and susceptible to wind transport (Lou et al., 2022). This finding indicates that SL and NT + XYY have the best protective effects. The vertical distribution characteristic pattern of soil particle size composition differs, SZ and NT + XYY, with the increase in soil depth, soil clay, powder, average particle size, and the gradual decrease in sorting coefficient. The decrease in fine particles with soil deepening is due to the ability of the edge-locked forest canopy to intercept wind-sand movement, resulting in the accumulation of fine particulate debris in the event of blocked settlement (Hupy, 2004). This finding is consistent with the study’s results by Dong et al. (2022). Tao and Zhang (2015) demonstrated that soil particle size fractal dimension can be used to reflect soil fertility status and the degree of soil exposure to wind and sand. The fractal dimension of soil particle size and mean particle size revealed positive connections with clay and silt concentration but negative associations with sand content (Ya et al., 2019). Similar findings were obtained in the present study (**Figure 5**), which indicated that fractal dimension and mean particle size could reflect the particle coarseness and fineness of the soil in the edge-locked forests, and fractal dimension increased with clayey and powdery grain content and decreased with sandy grain content. At the same time, higher soil silt and clay can keep SOC accumulating and increase the nitrogen input.

Furthermore, the characteristics of soil C, N, and P and their stoichiometric relationships are significantly influenced by soil texture (Wang et al., 2009). A correlation study found that SOC and TN had a substantial positive correlation with silt and clay but a negative correlation with sand. This is comparable to the findings of Tian et al., who concluded that soil texture significantly affects SOC and STN content (Tian et al., 2017) and that higher soil silt and clay can lead to the continual buildup of SOC (Wu et al., 2017). Plant production is stimulated by improving soil water-holding capacity and increasing soil C and N inputs. Soil C/N in the understory of the edge-locked forests was strongly and positively connected with powder grain content and considerably and negatively correlated with sand grain content, a finding contrary to the study of Marty et al. (2017). In comparison, the correlation of soil N/P with chalk and sand in the understory of the edge-locked forests was the opposite, suggesting that rough soil texture increases soil N/P.

## 5 Conclusions

In this paper, the following main conclusions were drawn through an in-depth analysis of the soil C, N, and P contents of six configuration types of edge-locked forests in the Kubuqi Desert, as well as their ecological stoichiometric characteristics and the vertical distribution characteristics of particle size.

1. The limiting nutrient element for the growth of edge-locked forests in the region is N. Of the 6 configuration types, the SOC and TN contents in the top 3 are SZ, NT + XYY, and SL. With the increase of soil depth, the soil C, N, C/P and N/P under the lock-edge forest gradually decreased, and the decrease of P content was not obvious. C/N showed the opposite pattern. and the phenomenon of epimerization of C and N contents was evident.

2. The area primarily comprised sand. The silt and clay contents of SL and NT + XYY are significantly higher than the other four configurations. Vertical distribution patterns of soil particle size varied under different configurations of edge-locked forests. SOC, TN, D, and *Mz* had substantial positive correlations with silt and clay concentration but negative correlations with sand content.

In summary, vegetation type, soil depth, and soil texture affect soil C, N, P, and stoichiometric characteristics. SZ and SL can be used as the dominant tree species in the Kubuqi Desert edge-locked forests, and the NT + XYY mixed forest configuration pattern has the most evident soil improvement effect.

## Data Availability

The original contributions presented in the study are included in the article/supplementary material, further inquiries can be directed to the corresponding author/s.
